# Book Review: *Eye-Tracking with Python and Pylink* by Zhiguo Wang

**DOI:** 10.1177/03010066221086083

**Published:** 2022-03-10

**Authors:** Edwin S. Dalmaijer

**Affiliations:** School of Psychological Science, University of Bristol

*Eye-Tracking with Python and Pylink* by Zhiguo Wang (2021) is one of those books that does exactly what it says on the cover. It provides a quick introduction to the Python programming language and to coding experiments with the PsychoPy or PyGame packages. The real strength of this book is in its extensive coverage of the Pylink library, a Python package produced by SR Research for its EyeLink eye-trackers. You would be hard-pressed to find a more comprehensive tutorial and reference to getting started with Python and EyeLink.

If you’re buying this book for its introduction to running experiments in Python, you might come away a bit disappointed. The coverage is relatively short, quite shallow, and seems to serve as a reminder more than anything else. The same is true for the chapters on experimentation with PsychoPy and PyGame, which introduce their basic syntax without much depth.

That said, the basics are not what this book is for. This book is about Pylink, and it *really* delivers on that promise. After covering the basics of eye tracking, the author dedicates several chapters on how to construct experiments using the Pylink library, and then on how to analyze the produced EyeLink Data Format (EDF) files. This includes advanced functionality that few other guides cover, like how to draw graphics or send TTL pulses on the host PC (the recording computer). Despite having used Pylink for almost a decade and having spent numerous hours reading through its documentation, I genuinely learned new things from these chapters.

During the experimentation chapters, readers are primed for later analyses with DataViewer, proprietary software from SR Research to process gaze data. The book explains what types of log messages are necessary to include in their data files to make the analysis as smooth as possible. The book extensively covers Pylink's functions, and explains how to set up a custom graphics class that allows readers to use EyeLink with their package of choice for psychology experiments (e.g. PsychoPy).

The chapter on data analysis explains the EDF format, the required conversion tool, and ways to process gaze and pupil data in Python scripts. This chapter covers many of the types of analyses and visualizations that one would conduct with gaze and pupil data, but it does so without going into too much detail on the required libraries (NumPy, Matplotlib, Pandas, Python Imaging Library). It echoes the introductory chapters in that it covers the basics very well, but nowhere near as comprehensively as its coverage of the Pylink library.

The book is written in an engaging way: it covers complex details without being overly technical, and it does so with a personable and to-the-point style without unnecessary frills. The author has opted to provide background information and specific explanations for particular code in the main text. This is alternated with extensive code listings that offer Python scripts that can be readily used by readers, with minor changes if necessary. Guidance on specific lines of code in these listings is provided as code comments within the listings themselves. This format makes for a slightly disjointed narrative structure (with explanations spread between main text and code listings), but it does ensure that code and explanation appear close together.

The only downside of this book is that its scope is rather narrow. While very popular, EyeLink is not the only system on the market. In addition, there are other tools than Pylink that can help researchers create experiments for EyeLink and other eye-trackers, including graphical experiment builders OpenSesame (Mathôt et al., 2012) and PsychoPy (Peirce et al., 2019); and general eye-tracking package PyGaze (Dalmaijer et al., 2014). Finally, there are better introductions to creating psychological experiments in Python and Python-associated software. If you’re desperate for a book, try Dalmaijer’s (2017) *Python for Experimental Psychologists* (disclaimers: the first edition was aimed at the now-defunct Python 2, and I am its author) or Peirce et al.’s (2022) *Building Experiments in PsychoPy* (focusses more on graphical interface than on code). There are also many online tutorials, which are free and usually more up-to-date than books.

In sum, this book is the best available reference material on using Python and Pylink to create experiments for eye-tracking with EyeLink devices. I would recommend this book even over SR Research's own documentation materials. If you’re looking for a general introduction to Python for psychological experiments, though, this might not be the book for you. However, if you own an EyeLink and would like to use Python and Pylink to do experiments, this book is an absolute must-have.
